# Comparison of Clinical Results and Pathological Examinations Between Locally Synthesized Bone-derived Hydroxyapatite and Medpor® Orbital Implants in Animal Models

**DOI:** 10.7759/cureus.3954

**Published:** 2019-01-25

**Authors:** Muji Roslinah, Wan-Hazabbah Wan Hitam, Md Salzihan Md Salleh, Suzina Syeikh Abdul Hamid, Ismail Shatriah

**Affiliations:** 1 Ophthalmology, School of Medical Sciences, Universiti Sains Malaysia, Kubang Kerian, MYS; 2 Pathology, School of Medical Sciences, Universiti Sains Malaysia, Kubang Kerian, MYS; 3 Otolaryngology, School of Medical Sciences, Universiti Sains Malaysia, Kubang Kerian, MYS

**Keywords:** bovine bone derived hydroxyapetite implant, commercially available porous polyethylene (medpor®) implant, animal models

## Abstract

We aimed to compare clinical and pathological reactions towards locally synthesized bovine bone derived from hydroxyapatite (bone docosahexaenoic acid (dHA)) and commercially available porous polyethylene (Medpor®, Porex Surgical Incorporation, Georgia, USA) orbital implants in animal models. An experimental study was performed on 14 New Zealand white rabbits. Group A (n=7) was implanted with bovine bone dHA and group B (n=7) was implanted with Medpor®. Clinical examinations were performed on Days 1, 7, 14, 28, and 42 post-implantation. The implanted eyes were enucleated on Day 42 and were sent for pathological evaluation. Serial clinical examinations included urine color and odor; feeding and physical activity demonstrated normal wellbeing in all the subjects. Localized minimal infection was observed in both groups during the first two weeks following implantation, and the subjects responded well to topical moxifloxacin. Both groups exhibited evidence of wound breakdown. No signs of implant migration or extrusion were observed in either group. The histopathological examination revealed no statistically significant difference in inflammatory cell reactions and fibrovascular tissue maturation between both types of implants. However, all (100%) of the bovine bone dHA implants displayed complete fibrovascular ingrowth compared to Medpor® implants (57.1%) at six weeks post-implantation (p=0.001). In conclusion, bovine bone dHA and Medpor® orbital implants were well-tolerated clinically and displayed similar inflammatory reactions and fibrovascular tissue maturation. Locally synthesized bovine bone dHA orbital implants displayed significantly greater complete fibrovascular ingrowth in comparison with Medpor® implants.

## Introduction

The use of orbital implants to replace lost volume was a major breakthrough in anophthalmic socket surgery. The implant aims to reduce frequent incidences and severity of infection due to the availability of immune responses, allowing soft tissue connection between the extraocular muscles and resulting in better motility and reduced implant migration and extrusion [1]. The most common porous orbital implants used today include hydroxyapatite, porous polyethylene, and aluminum oxide.

Bone derived from hydroxyapatite (bone dHA) orbital implants was first introduced by Schmidt in 1899. Jordan et al. documented that this implant had pore sizes that varied from 300 to 600 µm [2]. Pore size appears to have an effect on the rate of fibrovascular ingrowth, which occurs more rapidly in implants with a 200 µm pore size than in implants with a 500 µm pore size [3]. Porous polyethylene implants were introduced over a decade ago and have been available for orbital implantation (Medpor®, Porex Surgical Incorporation, Georgia, USA) since 1991. They have pores similar to hydroxyapatite but are less uniform in size and more irregular in shape. Commercially available Medpor® has pore sizes of 100-500 μm and 125-1000 μm.

In current practice, Medpor® has been accepted as the preferred option of orbital implant in Malaysia. However, its cost is still the most concerning drawback. The National Tissue Bank of Universiti Sains Malaysia has invented and prepared biocompatible bovine bone dHA orbital implants at a lower cost [4]. We conducted this study in order to compare clinical and pathological reactions toward locally synthesized bovine bone dHA and Medpor® orbital implants in animal models.

## Materials and methods

This experimental study was conducted at the Laboratory of Animal Research and the Laboratory of Histopathology, School of Medical Sciences, Universiti Sains Malaysia, Kubang Kerian, Kelantan, Malaysia. Approval was obtained from the Research and Ethical Committee, School of Medical Sciences, and Animal Ethics Committee, Health Campus, Universiti Sains Malaysia. The study’s protocol adhered to the Association for Research and Vision in Ophthalmology Statement for the Use of Animals in Ophthalmic and Vision Research.

Fourteen healthy New Zealand white rabbits weighing between two and three kg were included in this study. All animals were free from any eye disease and were kept, fed, and cared for in the Laboratory Animal Research, Universiti Sains Malaysia, Kubang Kerian, Kelantan, Malaysia.

These rabbits were randomly divided into two groups (A and B) using single block randomization. Group A subjects (n=seven) were implanted with the bovine bone docosahexaenoic acid (dHA) while group B subjects (n=seven) were implanted with Medpor®. The implantation was performed in the right eye of each rabbit. All the rabbits that had successful implantation were included in the study. Rabbits were excluded if the implantation failed or if severe systemic infection developed following the implantation phase.

**Figure 1:**
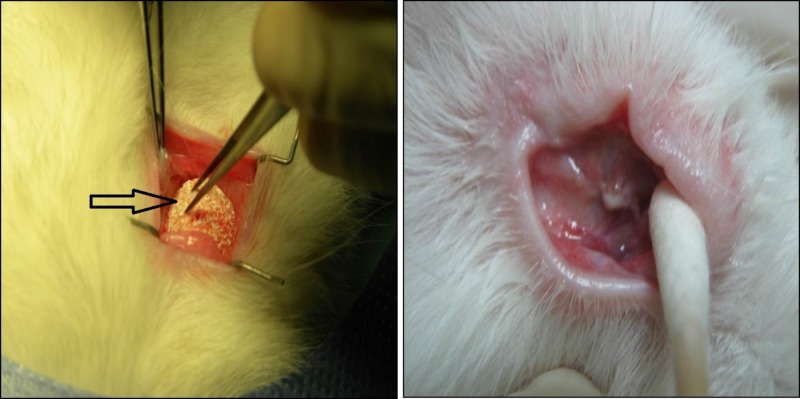
Implantation of bovine bone dHA implant (black arrow) in rabbit’s eye (left photo). Well sutured conjunctiva with the implant in-situ (right photo)

The bovine dHA implants were synthesized from local bovine femoral heads and prepared at the National Tissue Bank, Universiti Sains Malaysia, Kubang Kerian, Kelantan, Malaysia [4]. Medpor® implants were supplied by the manufacturer. They were implanted after evisceration was performed on the right eye by an identified investigator (Figure 1).

Serial clinical examinations were performed on Days 1, 7, 14, 28, and 42 post-implantation by another masked investigator. The general wellbeing of the animals was assessed based on the presence of eye discharge, urine color and odor, feeding, and physical activity. The wounds were examined using a portable slit lamp for integrity, implant migration, or extrusion. On Day 42, the rabbits underwent euthanasia and the eyes with the implants were enucleated.

The orbital implants together with scleral coating were dissected and maintained in a 4% neutral formalin buffered solution. The gross appearances of the bisected implants were examined by an assigned independent examiner. The specimens were then prepared using the undecalcified method. After the fixation process, the samples were dehydrated and then embedded in a methacrylate solution (Technovit 720 Virus Creation Laboratory, Kulzer, Wehrheim, Germany).

After polymerization, they were processed according to the cutting-grinding technique and sliced between 0.1 and 0.3 mm thick [5]. The sections were stained with hematoxylin and eosin and examined using light microscopy. They were evaluated for the grade of cellular inflammation and fibrovascular ingrowth according to standardized histopathological grading as described by Tienen et al. and Chung et al. [6-7]. The findings are summarized in Tables 1-2.

**Table 1:** Grading of fibrovascular tissue maturation

Grade	Definition
1	Edematous young granulation tissue with acute inflammatory cells infiltration. Initial stage of capillary vessels and fibroblast growth.
2	Decreasing tissue edema with chronic inflammatory cell infiltration and many capillary vessels. Initial stage of fibroblast proliferation.
3	Maturation of fibroblast and many capillary vessels. Initial stage of collagen fibers growth.
4	Minimal tissue edema with decreased capillary vessels. Collagen fibers maturation.
5	Most of granulation tissue composed of collagen fibers.

**Table 2:** Grading of inflammatory cell reaction

Grade	Definition
1	No inflammation
2	Slight inflammation with many macrophages and giant cells
3	Well-defined inflammation with many macrophages and giant cells but no polymorphonuclear (PMN) leucocytes
4	Moderate inflammation as grade 2 but with few PMN leucocytes
5	Severe inflammation, abundant macrophages, giant cells, and PMN leucocytes

Statistical analysis was performed with the Statistical Package for the Social Sciences (SPSS) version 11.0 (IBM Corp, Armonk, NY, US). Data were analyzed by a statistical test with statistical significance set at p<0.05.

## Results

Clinical examination

In both groups, eye discharge was observed as early as Day 1 post-implantation. Three subjects (42.9%) emanated from the bovine bone dHA group and two subjects (28.6%) were derived from the Medpor® group. Conjunctival swab culture and sensitivity proved negative in all the involved subjects. They were treated with topical moxifloxacin four times per day in the affected eyes. All subjects had normal urine color, urine odor, and physical and feeding activity throughout the study period. The results are shown in Table 3.

**Table 3:** Serial clinical findings Bone dHA refers to bone derived hydroxyapatite

Parameter		Bone dHA group n=7 (%)	Medpor® group n=7 (%)
Presence of eye discharge			
Day 1	Yes	2 (28.6)	3 (42.9)
	No	5 (71.4)	4 (57.1)
Day 7	Yes	2 (28.6)	2 (28.6)
	No	5 (71.4)	5 (71.4)
Day 14	Yes	2 (28.6)	2 (28.6)
	No	5 (71.4)	5 (71.4)
Day 28	Yes	0 (0.0)	0 (0.0)
	No	7 (100.0)	7 (100.0)
Day 42	Yes	0 (0.0)	0 (0.0)
	No	7 (100.0)	7(100.0)
Urine color and odor			
	Normal	7 (100.0)	7 (100.0)
	Abnormal	0 (0.0)	0 (0.0)
Feeding activity			
	Normal	7 (100.0)	7 (100.0)
	Abnormal	0 (0.0)	0 (0.0)
Physical activity			
	Normal	7 (100.0)	7 (100.0)
	Abnormal	0 (0.0)	0 (0.0)

All subjects from both groups displayed no implant migration or extrusion. The bovine bone dHA group demonstrated partial wound integrity earlier than the Medpor® group. One subject from the bovine bone dHA group exhibited changes on Day 7 while two subjects from the Medpor® group displayed partial wound integrity during Day 14 of observation. These three subjects demonstrated partial wound integrity until the last observation day.

Gross and microscopic histopathological examination

**Figure 2:**
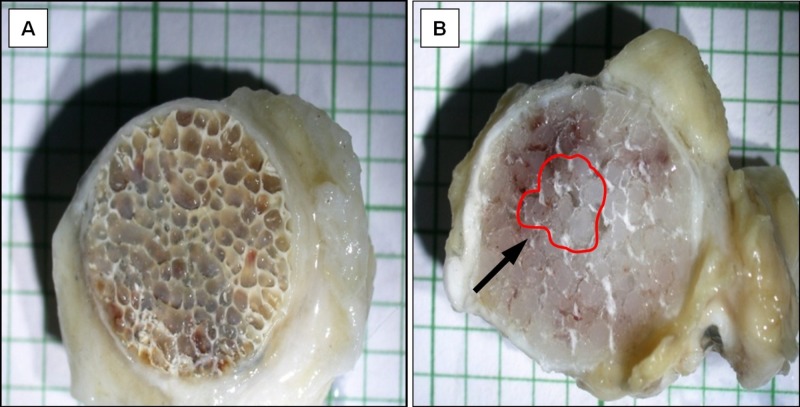
Cross section. (A) bovine bone dHA implant showing tissue ingrowth occupying the entire implant area. (B) Medpor® implant showing tissue ingrowth sparing the central area (black arrow) of the implant

All specimens displayed visible tissue ingrowth in between interconnecting channels from the peripheral towards the center of the implant. Specimens from the Medpor® group demonstrated a relatively smooth implant surface. Their interconnecting channels were more uniform and noticeably smaller in size than the bovine bone dHA implants. Tissue ingrowth was observed in the entire bovine bone dHA implant, whilst the central-most area was spared in the Medpor® implant (Figure 2).

Grade 1 inflammatory cell reaction was observed in two (28.6%) specimens with bovine bone dHA implants but none in the specimens with the Medpore® implant. Four (57.1%) specimens demonstrated Grade 2 inflammatory reactions in both groups. The remaining specimens exhibited a Grade 3 inflammatory reaction. No specimen disclosed Grade 4 or Grade 5 inflammatory reactions. However, there occurred no significant differences between both groups (p=0.560, Chi-square test). The results are shown in Tables 4-5.

**Table 4:** Grade of inflammatory cell reaction Bone dHA refers to bone derived hydroxyapatite

Implants	Inflammatory cell reaction				
	Grade 1 n (%)	Grade 2 n (%)	Grade 3 n (%)	Grade 4 n (%)	Grade 5 n (%)
Bone dHA	2 (28.6)	4 (57.1)	1 (14.3)	0 (0.0)	0 (0.0)
Medpor®	0 (0.0)	4 (57.1)	3 (42.9)	0 (0.0)	0 (0.0)

**Table 5:** Comparison of inflammatory cell reaction Bone dHA refers to bone derived hydroxyapatite

	Implant		*p-value
	Bone dHA n=7 (%)	Medpor® n=7 (%)	
Low grade (Grade 1-2)	6 (85.7)	4 (57.1)	0.560
High grade (Grade 3-5)	1 (14.3)	3 (42.9)	

Both groups demonstrated fibrovascular tissue maturation after six weeks, as described in Tables 6-7.

**Table 6:** Grade of fibrovascular tissue maturation Bone dHA refers to bone derived hydroxyapatite

Implants	Fibrovascular tissue maturation				
	Grade 1 n (%)	Grade 2 n (%)	Grade 3 n (%)	Grade 4 n (%)	Grade 5 n (%)
Bone dHA	0 (0.0)	0 (0.0)	0 (0.0)	2 (28.6)	5 (71.4)
Medpor®	0 (0.0)	1 (14.3)	1 (14.3)	2 (28.6)	3 (42.9)

**Table 7:** Comparison of fibrovascular tissue maturation Bone dHA refers to bone derived hydroxyapatite

	Implant		*p-value
	Bone dHA n=7 (%)	Medpor® n=7 (%)	
Low grade (Grade 1-2)	0 (0.0)	2 (28.6)	>0.950
High grade (Grade 3-5)	7 (53.8)	5 (71.4)	

**Figure 3:**
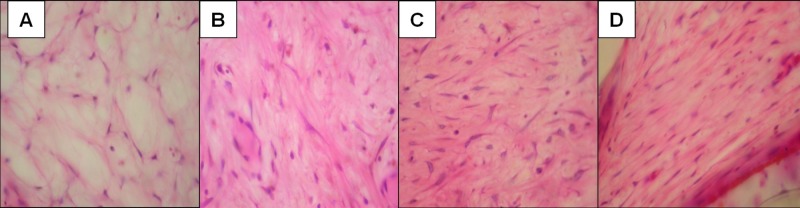
Grade of fibrovascular ingrowth maturity. (A) Grade 2: Fibrovascular maturation with decreasing tissue edema, many capillary vessels and initial stage of fibroblast proliferation, (B) Grade 3: Fibrovascular maturation with maturation of fibroblast and many capillary vessels, (C) Grade 4: Fibrovascular maturation with minimal tissue edema and decreased capillary vessels, (D) Grade 5: Fibrovascular maturation, most of granulation tissue composed of collagen fibers

**Figure 4:**
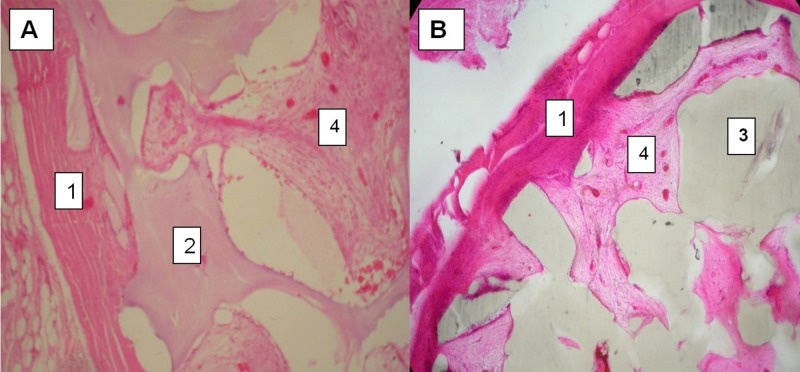
Fibrovascular ingrowth in between pores in bone dHA (A) and Medpor® (B) implants (1=scleral coat, 2=bovine bone dHA material, 3=Medpor® material, 4=fibrovascular tissues) dHA: docosahexaenoic acid

Grade 4 maturation was observed in two (28.6%) specimens from the bovine bone dHA and Medpore® groups, respectively. Grade 5 maturation was displayed by five (71.4%) specimens from the bovine bone dHA group and three (42.9%) from the Medpore® group. However, there was no statistically significant difference between the groups (p>0.950). Figures 3-4 demonstrate evidence of fibrovascular tissue maturation in both groups.

Tables 8-9 present the depth of fibrovascular ingrowth at six weeks between the studied groups.

**Table 8:** Grade of depth of fibrovascular ingrowth Bone dHA refers to bone derived hydroxyapatite

Bone dHA	Depth (%)	Medpor®	Depth (%)
1	100.0	1	45.5
2	100.0	2	82.1
3	100.0	3	90.2
4	100.0	4	73.2
5	100.0	5	86.8
6	100.0	6	66.5
7	100.0	7	88.4

**Table 9:** Comparison depth of fibrovascular ingrowths Bone dHA refers to bone derived hydroxyapatite

	Implant		*p-value
	Bone dHA	Medpor®	
	Median (IQR)	Median (IQR)	
Depth	100 (0.0)	75.7 (16.0)	0.001

All specimens from the bovine bone dHA group achieved 100% depth of fibrovascular ingrowth while none from the Medpor® group displayed similar features. One specimen from the Medpor® group demonstrated evidence of ingrowth less than 50% in depth. Statistical analysis exemplified a significant difference between both groups (p=0.001).

## Discussion

Our study demonstrated that all subjects from the bovine bone dHA group had similar clinical reactions as those in the Medpor® group. No cross-infection was demonstrated in either group. Subclinical infection, as evidenced by the presence of eye discharge that responded to a topical antibiotic, was demonstrated in five (35.7%) subjects.

The above observation is consistent with an animal study reported by Sclafani et al. [8]. They implanted expanded polytetrafluoroethylene and Medpor® in Sprague-Dawley rats. Staphylococcus aureus was inoculated 14 days after implantation to induce infection, and 75.0% of the implants remained clinically uninfected. They suggested that the presence of vascularized host tissue in and around the implant lends resistance to experimentally induced infection. Recently, Fernandez-Bueno et al. reported a similar outcome between Medpor® and a new porous implant (Oculfit I and II) [9].

Our study showed that bovine bone dHA implants developed early wound dehiscence. We postulated that the rough surface of the bovine bone dHA contributed to this early complication. In contrast, two subjects from the Medpor® group developed wound dehiscence much later on Day 14 post-implantation. The smoother surface of the Medpor® implant caused less friction, thereby reducing the rate of wound dehiscence. This suggests that the Medpor® implant has better wound integrity than the bovine bone dHA implant. However, we are unable to proceed with appropriate statistical tests.

Our results are consistent with findings reported by Goldberg et al. They described six cases of conjunctival dehiscence with a hydroxyapatite implant at a mean of 4.5 weeks post-implantation [10]. They hypothesized that spicules of the implant inhibited epithelialization, which subsequently led to wound dehiscence.

Kim et al. also reported a similar observation [11]. They compared 32 subjects with orbital implants and reported three cases of wound dehiscence that included two cases of hydroxyapatite implants and one case of a Medpor® implant. The low rate of wound dehiscence with Medpor® implants is consistent with other previously reported studies [12-14].

 We noted that the bovine bone dHA group demonstrated a higher trend of tissue maturation. Grade 5 fibrovascular maturation was seen in 71.4% compared to 42.9% of subjects in the Medpor® group. However, these data were not statistically significant.

We observed that fibrovascular ingrowth developed in the centripetal progression toward the center of the implants. All of the bovine bone dHA groups showed complete fibrovascular ingrowth while only 57.1% from the Medpor® group demonstrated more than 80% depth of ingrowth towards the end of the six-week period. Our observation is parallel with a few other published studies, which reported that complete fibrovascularization occurred after the Medpor® implant was observed at 12 weeks [15-19].

Hsu et al. implanted the Medpor® implant into 12 rabbits and reported that the extent of fibrovascular ingrowth at six weeks was at an average of 76.3%. This is lower than our findings of an average of 82.9% [16]. Rubin et al. compared the extent of fibrovascular ingrowth in porous polyethylene versus hydroxyapatite [18]. They reported complete fibrovascularization at six weeks in hydroxyapatite and 12 weeks in porous polyethylene. 

The size and distribution of the pores in the porous implant determined the rapidity of fibrovascular ingrowth. Generally, larger pore size will allow more rapid fibrovascularization. Our study demonstrated how the bovine bone dHA implant achieved complete fibrovascularization after six weeks. This is in keeping with gross specimen observation, which displays larger pore sizes (300-600μm) in bone dHA than in Medpor® (100-500μm). However, previous studies reported that fibrovascular ingrowth was sluggish when pore sizes were larger than 700 μm [17-18,20]. These were attributed to an insufficient supporting structure, increased fragility, decreased intensity, and higher risk of infection [17-18,20].

We confirmed fibrovascular ingrowth with histopathological examinations in our study. In clinical practice, this method would be impossible. There were reported studies that advocated the use of post-contrast magnetic resonance imaging (MRI) in assessing fibrovascular ingrowth in porous orbital implants [21-22]. They reported that the MRI study was identical to the histological fibrovascular ingrowth pattern [21-22].

There are two main drawbacks in our experimental animal study. We were limited to a small sample size and a short observation period. Perhaps a bigger sample size and a longer observational period would have more advantages and describe a better picture.

## Conclusions

Locally synthesized bovine bone dHA orbital implants were as well-tolerated clinically as commercially prepared Medpor® implants in our animal models. All bovine bone dHA displays complete fibrovascular ingrowth as compared to 57.1% of Medpor® implants at six weeks post-implantation. However, both implants demonstrated a similar degree of inflammatory cell reaction and fibrovascular tissue maturation.
